# Sodium content of packaged foods and beverages in Nigeria: a cross-sectional comparative study with WHO global sodium benchmarks, Kenya, and South Africa

**DOI:** 10.1017/jns.2025.10076

**Published:** 2026-03-04

**Authors:** Adedayo E. Ojo, Alexandra Jones, Kylie Howes, Vanessa Alfa, Anthony Orji, Oluwafemi A. Stephen, Carla Pool, Alex Kibet, Fraser Taylor, Diederick E. Grobbee, Bruce Neal, Mark D. Huffman, Dike B. Ojji, Sanne A. E. Peters

**Affiliations:** 1 Cardiovascular Research Centre, University of Abuja Teaching Hospital, University of Abuja, Abuja, Nigeria; 2 Julius Center for Health Sciences and Primary Care, University Medical Center, Utrecht University, Netherlands; 3 George Institute for Global Health, University of New South Wales, Sydney, Australia; 4 Food and Drug Services Department, Federal Ministry of Health and Social Welfare, Abuja, Nigeria; 5 Discovery Vitality, Sandton 2146, South Africa, South Africa; 6 Kenya Medical Training College, Kenya; 7 School of Public Health, Imperial College London, UK; 8 Cardiovascular Division and Global Health Center, Washington University, St. Louis, USA; 9 Northwestern University, Chicago, USA; 10 Cardiovascular Research Unit, University of Abuja Teaching Hospital, Nigeria; 11 Department of Medicine, Faculty of Clinical Sciences, University of Abuja, Nigeria; 12 The George Institute for Global Health, Imperial College, UK; 13 Julius Center for Health Sciences and Primary Care, University Medical Center Utrecht, Netherlands

**Keywords:** Cardiovascular disease, Hypertension, Nigeria, Packaged foods, Sodium, WHO benchmarks

## Abstract

High intake of processed foods, especially those with high sodium content, is a contributor to hypertension and cardiovascular disease. This study aimed to compare the sodium content of packaged foods and beverages in Nigeria to WHO Global Sodium Benchmarks and similar products in Kenya and South Africa. The study examined packaged foods from major retail stores in the capital cities of the Federal Capital Territory, Kano, and Ogun states in Nigeria from November 2020 to March 2021. Benchmark values were based on the 2021 WHO Global Sodium Benchmarks. We used secondary data from packaged food surveys conducted in South Africa (2015, 2016 and Kenya 2019). Approximately 40.0% (*n* = 36) of subcategories of packaged foods were captured in the WHO global sodium benchmark. Of these, 64.0% (*n* = 23) exceeded the benchmarks, including ‘processed meat’ (912.0 vs. 250.0 mg/100 g), cheese (776.0 vs. 190 mg/100 g), and ‘wholegrain chips’ (930.0 vs. 470 mg/100 g). Exactly 36.0% (*n* = 13) had lower sodium content, such as ‘rice-based snacks’ (113.0 vs. 520 mg/100 g) and ‘dried seafood’ (400 vs. 800 mg/100 g). In seven out of eleven main food categories (64%), Nigeria had a higher sodium content compared to Kenya. Similarly, Nigeria exhibited higher sodium content than South Africa in six out of eleven food categories (55.0%). With 64.0% of Nigerian subcategories exceeding WHO benchmarks and higher sodium levels than South Africa and Kenya in most categories. These findings highlight the urgent need for targeted sodium reduction and product reformulation to align Nigeria’s packaged foods with international benchmarks.

## Background

High dietary intake of processed foods, particularly those high in sodium, is a significant risk factor for various adverse health outcomes, including hypertension and CVDs.^(1,2)^ According to the Global Burden of Disease Study, CVDs remain the leading cause of death globally.^(3)^ In 2019, there were approximately 523.2 million individuals with CVD globally; this encompasses a range of conditions, including coronary artery disease, stroke, heart failure, and other related cardiovascular disorders.^(4)^ The WHO recommends dietary sodium reduction as one of the best buys to improve global cardiovascular health.^(5,6)^ Currently, an increasing number of countries are developing and executing strategies aimed at reducing sodium intake.^(7–9)^ These strategies include reformulating food supplies, implementing front-of-package labelling, employing taxation measures, conducting consumer education campaigns, and implementing interventions to enhance food procurement policies for public institutions.^(10–13)^

The WHO has set Global Sodium Benchmarks as part of its efforts to promote public health and reduce the burden of CVDs.^(14,15)^ The global benchmarks serve as a tool to encourage the reformulation of packaged food products, thereby driving progress in sodium reduction efforts. They complement the existing national and regional initiatives in setting sodium targets and play a vital role in guiding countries to develop policies and strategies at the national level.^(16,17)^ The WHO Global Sodium Benchmarks are consistent with Nigeria’s 2019 National Multi-Sectoral Action Plan (NMSAP) for the prevention and control of Noncommunicable Diseases (NCDs),^(18)^ which incorporates policies derived from the WHO SHAKE package. The priority actions and strategies outlined in the WHO Surveillance, Harness industry, Adopt standards for labelling and marketing, Knowledge (SHAKE) package encompass mandatory sodium limits in processed foods, advertising restrictions, mass-media campaigns, school-based interventions, and enhanced front-of-package labelling.^(19)^

In Nigeria, like many other low- and middle-income countries (LMICs), there has been a notable transition in dietary habits, characterised by a rising consumption of processed and packaged foods.^(20)^ The high sodium consumption in Nigeria is closely linked to the prevalence of hypertension, which affects an estimated 25% to 40% of Nigerian adults.^(21)^ Available studies suggest that average dietary salt intake in Nigeria is between 7–10 g/day, which exceeds the WHO recommended maximum of 5 g/day.^(22,23)^ Discretionary sources, including added salt and bouillon cubes, are believed to contribute the largest share of sodium intake,^(24)^ while processed and packaged foods are an increasingly important contributor in urban areas.^(25–26)^ However, nationally representative data on the relative contribution of discretionary and non-discretionary sodium intake in Nigeria remain limited, and further research is needed to fill this gap. The 2022 Pre-Packaged Food Labelling Regulations^(27)^ of the National Agency for Food and Drug Administration and Control (NAFDAC) outline the requirements for food labelling. Under these regulations, nutrition information panels, including sodium content, are mandatory when nutrition or health claims are made or when the food is intended for special dietary use. However, mandatory declaration of sodium content on all packaged foods has not yet been fully implemented.

In South Africa, nutrition labelling is regulated under the Regulations Relating to the Labelling and Advertising of Foodstuffs,^(28)^ which mandates nutrition information panels when a nutrient content claim is made or when a food is fortified. While sodium content labelling is not mandatory for all foods, it is required when relevant claims are made, and the country has implemented mandatory maximum sodium limits for certain processed food categories since 2016. In Kenya, nutrition labelling is guided by the 2018 Food, Drugs and Chemical Substances Food Labelling, Advertising and Standards Regulations,^(28)^ which require nutrition information when a nutrient content claim is made or for foods intended for special dietary uses; although sodium content labelling is not yet mandatory for all packaged foods, voluntary provision is encouraged, and front-of-pack labelling guidelines have been proposed. These countries therefore provide useful regional comparators for Nigeria.

Utilising data from the Nigeria Sodium Study (NaSS), the current study explores sodium levels in commercially packaged foods and beverages, comparing these levels with WHO benchmarks to generate valuable evidence for policymaking and sodium reduction strategies in Nigeria. Additionally, the study extends its scope by comparing sodium content in Nigerian packaged foods with similar studies conducted in South Africa and Kenya. This comparative analysis is crucial for understanding regional variations in sodium consumption and informing public health strategies tailored to the African context. South Africa and Kenya were selected for comparison due to their prior studies on sodium content in packaged foods, allowing for a meaningful evaluation across different regulatory and market environments. South Africa, having implemented sodium reduction policies, provides insight into the impact of regulatory measures, while Kenya, representing an East African perspective, offers a contrast in a non-regulatory context. By incorporating these comparisons, this study aimed to provide a critical regional assessment of sodium levels in packaged foods, strengthening the evidence base for sodium reduction initiatives in Nigeria and contributing to broader public health efforts across Africa.

## Methods

### Data collection

Data for Nigeria were collected between November 2020 and March 2021 as part of the NaSS using the FoodSwitch data collection protocol.^(32)^ This involved in-store surveys of major retail supermarkets, convenience stores, and other outlets in the Federal Capital Territory, Kano, and Ogun states to capture a representative range of commercially packaged foods and beverages available in the market. Data were collected from major supermarkets and retail outlets in Nigeria, which represent the largest proportion of the formal food retail market and capture the majority of available pre-packaged products in urban areas. However, precise market share estimates of these outlets are not publicly available. In Nigeria, nutrition information panels, including sodium content, are mandatory only when a health or nutrition claim is made or when the product is for special dietary use; for other packaged foods, nutrition declarations remain voluntary. These requirements are outlined under the NAFDAC Pre-Packaged Food Labelling Regulations.^(27)^ Product information, including the Nutritional Information Panel (NIP), was photographed and recorded for all eligible packaged products found during the survey period. The South African dataset was obtained from the 2015–2016 FoodSwitch database, and the Kenyan dataset from 2018–2019, both of which were compiled using similar in-store survey protocols.^(30,31)^ Only products with a declared sodium value on the nutrition information panel were included in the main analysis.

### Data extraction

During data collection, we captured the presence or absence of a partial or complete nutrient declaration on pack. Where a nutrient declaration was present, we extracted available information on the following nutrients, converting values where necessary into standardised measurements: energy (kJ/100 g), protein (g/100 g), saturated fat (g/100 g), trans fat (g/100 g), carbohydrate (g/100 g), total sugar (g/100 g), sodium (mg/100 g), and salt (g/100 g). All nutrient values were standardised into consistent units (mg/100 g). For products where sodium was reported as salt (NaCl), salt values were converted to sodium and expressed as mg/100 g by multiplying the salt value by 400. We defined a partial nutrient declaration as including information on at least one of the components specified in the Nigerian regulation, and a complete nutrient declaration as providing information on all components specified in the 2019 Nigerian regulation, i.e., energy, saturated fats, trans fats, carbohydrates, total sugars, protein, and either sodium or salt. We also extracted information on product manufacturers. All extracted data were checked for completeness and consistency. Duplicate products and implausible values were removed, and sodium values were cross-validated where both sodium and salt were declared. Units were standardised across all products. To ensure accuracy, a random 10% sample of extracted entries was independently verified by a second researcher, and discrepancies were resolved. The procedures for data entry and quality assurance for the databases have been previously outlined and were consistently applied across all three countries.^(32)^

### Food categorisation

The studies conducted in Nigeria, Kenya, and South Africa applied similar food categorisation systems. Products were classified using the hierarchical structure developed by the Global Food Monitoring Group (GFMG),^(33)^ which organises foods and beverages into broad groups and further into specific categories for consistent global comparison. Trained dietitians and nutritionists assigned products to 16 primary food groups: (a) bread and bakery items, (b) cereal and grain products, (c) confectionery, (d) convenience foods, (e) dairy, (f) edible oils and oil emulsions, (g) alcohol, (h) specialised dietary foods, (i) fruits, vegetables, nuts, and legumes, (j) meat and meat alternatives, (k) non-alcoholic beverages, (l) sauces, dressings, spreads, and dips, (m) seafood and seafood products, (n) snack foods, (o) sugars, honey, and related items, and (p) vitamins and supplements. Each product was identified by a unique barcode.^(32,34)^ For comparison with the WHO Global Sodium Benchmarks, Products were initially classified using the GFMG system, which contains more categories than the WHO Global Sodium Benchmark framework. Only subcategories that were comparable to WHO Global Sodium Benchmark categories were included in the analysis. For each product, sodium content was compared with the corresponding WHO benchmark, and the proportion of products above and below the thresholds was calculated. We also compared the category median sodium content against the WHO benchmarks and reported the percentage deviation. The NaSS study applied rigorous quality assurance measures, including cross-checking at least 10% of products for correct classification, as described in the published study protocol. This approach aligns with FoodSwitch, an international initiative promoting healthier food choices through standardised food categorisation.^(24,28,29)^ For broad categories such as ‘miscellaneous cereals and grain products’, examples include semolina, wheat flour mixes, and maize flour. Similarly, ‘snack foods not otherwise specified’ included mixed packaged snack products that did not fit into predefined WHO benchmark categories.

### Statistical analysis

Descriptive statistics were computed to assess the sodium levels mg per 100 g for each subcategory.

For cross-country comparisons, data were analysed at the food category level. Median sodium values were used because sodium distributions were skewed, and these medians were compared across categories between Nigeria, Kenya, and South Africa. Sodium content distributions for each dataset were tested for normality using the Shapiro–Wilk test. The data were highly skewed, therefore sodium levels were summarised using medians and interquartile ranges. For cross-country comparisons, data were analysed at the food category level. Sodium content distributions were first tested for normality using the Shapiro–Wilk test, and because the data were not normally distributed, median values were used to summarise sodium levels. These medians were then compared across categories between Nigeria, Kenya, and South Africa.

Given the large number of statistical comparisons across food categories, we considered the potential for type I error. Although we did not formally adjust for multiple testing, our interpretation focused on overall trends and consistent patterns across categories rather than isolated significant results.^(35)^ In categories not covered by the WHO Global Sodium Benchmarks, we applied the 33rd percentile of sodium content as a reference value for defining targets. This approach has been recommended in the WHO South-East Asia Region Sodium Benchmarks and provides a practical and achievable threshold for product reformulation in the absence of global benchmarks.^(36)^ All analyses were conducted using R version 4.4.0 (2024-04-24 UCRT). No imputation was conducted for missing data. Statistical significance was defined as a two-sided *p* value < 0.001 without adjustment for multiple testing.

## Results

In Nigeria, information on packaged foods and beverages was gathered from retail establishments located in the Federal Capital Territory (16 outlets), Kano (17 outlets), and Ogun (12 outlets) state. A total of 7039 packaged foods and beverages from 1089 distinct manufacturers were identified. These products encompassed 16 major food groups and were classified into 89 different food categories. The survey conducted in Kenyan supermarkets identified 6003 distinct packaged foods and beverages within 56 different food categories. In South Africa, a total of 11,065 foods were collected within 57 food categories (Table 2).

### Nigeria sodium content and WHO global sodium benchmarks

**Figure 1.**
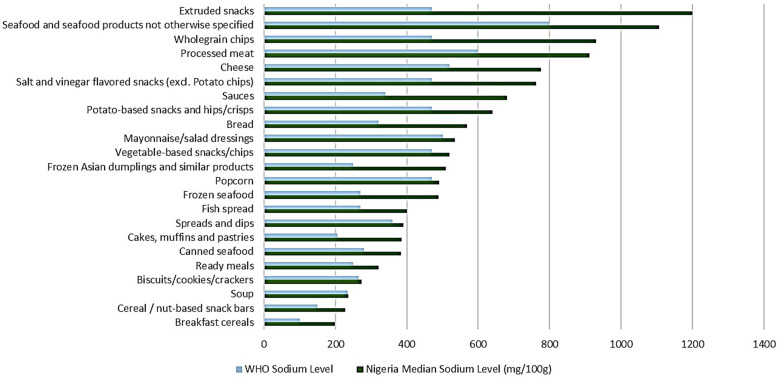
Sodium content of packaged foods and beverages in Nigeria compared with WHO Global Sodium Benchmarks (high-sodium products).

**Figure 2.**
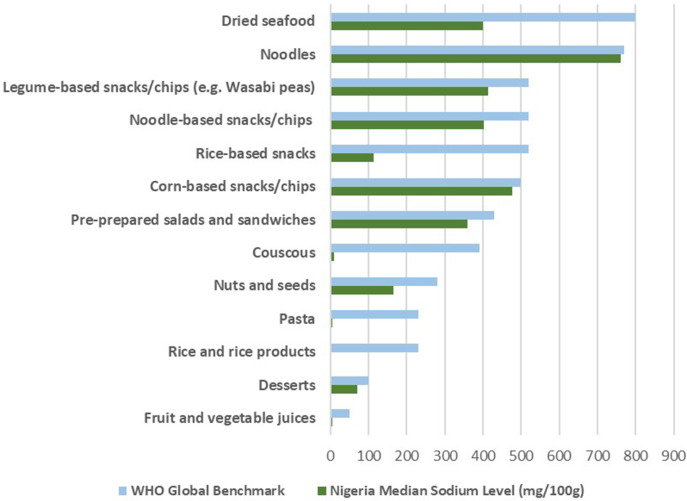
Sodium content of packaged foods and beverages in Nigeria compared with World Health Organization (WHO) sodium benchmarks (low-sodium products).

Among the Nigerian packaged food and beverage subcategories analysed, 40.0% (*n* = 36) corresponded to subcategories included in the WHO Global Sodium Benchmarks. Table 1 presents the comparison of sodium content by subcategories of packaged foods and beverages in Nigeria with the WHO Global Sodium Benchmarks. In the subcategories of Nigerian food products, the sodium content varied from 0.0 mg/100g to a maximum of 1200.0 mg/100g. Figures 1 and 2 compare the median sodium content of food and beverage categories in Nigeria with WHO benchmarks for high and low sodium content categories, respectively. A median sodium content above the global benchmark was observed in 64.0% (*n* = 23) of the subcategories.

**Table 1.** Comparison of sodium content by category of packaged foods and beverages in Nigeria and the WHO Global Sodium Benchmarks

Main food category	Subcategory	Total number of products	Sodium content			
			Median (mg/100 g)	IQR (mg/100 g)	WHO global benchmark (mg/100g)	*P*-value
Bread and bakery products	Biscuits/cookies/crackers	1240	273	186	265	**<0.001**
	Bread	20	569	1114	320	0.118
	Cakes, muffins and pastries	92	386	861	205	**<0.001**
Cereal and grain products	Breakfast cereals	373	198	387	100	**<0.001**
	Couscous	8	9	6	390	0.125
	Noodles	59	760	1342	770	<0.001
	Pasta	143	4	8	230	0.038
	Rice and rice products	74	2	12	230	<0.001
Confectionery	Chocolate and sweets/candy	592	96	116	760	<0.001
Convenience foods	Frozen Asian dumplings/similar products	3	509	262	250	0.500
	Ready meals	12	320	170	250	0.063
	Soup	27	236	68	235	0.625
Seafood and seafood products	Canned seafood	117	383	60	280	**0.044**
	Dried seafood	12	400	408	800	0.031
	Fish spread	2	400	160	270	0.250
	Frozen seafood	4	489	507	270	0.500
	Seafood and seafood products not otherwise specified	4	1107	980	800	0.500
Dairy	Cheese	49	776	360	520	**<0.001**
	Desserts	59	71	178	100	0.250
	Nuts and seeds	154	166	412	280	0.062
Non-alcoholic beverages	Fruit and vegetable juices	218	4	10	50	<0.001
Sauces, dressings, spreads and dips	Mayonnaise/salad dressings	153	535	385	500	0.220
	Sauces	367	680	974	340	**<0.001**
	Spreads and dips	88	391	448	360	**0.006**
	Processed meat	79	912	460	600	**<0.001**
Snack foods	Cereal/nut-based snack bars	35	228	250	150	**<0.001**
	Corn-based snacks/chips	20	476	291	500	0.309
	Extruded snacks	1	1200	0	470	**<0.001**
	Legume-based snacks/chips (e.g. Wasabi peas)	9	414	633	520	0.625
	Noodle-based snacks/chips	3	401	621	520	0.591
	Popcorn	37	491	699	470	0.264
	Potato-based snacks and hips/crisps	137	640	326	470	**<0.001**
	Pretzels	2	850	100	760	0.500
	Rice-based snacks	12	113	485	520	<0.001
	Salt and vinegar flavoured snacks (excl. Potato chips)	7	763	719	470	0.438
	Vegetable-based snacks/chips	9	520	343	470	0.594
	Wholegrain chips	2	930	340	470	0.749

**Table 2.** Characteristics of packaged food and beverage products collected in Nigeria, Kenya, and South Africa

Country	Year of data collection	No. of stores sampled	No. of products collected	No. of food categories represented
Nigeria	2020–2021	45	7039	89
Kenya	2019	64	6003	56
South Africa	2015–2016	Major supermarket chains (Shoprite, Pick n Pay, Spar, Woolworths)	11065	57

Significant differences were observed across several food subcategories, including biscuits/cookies/crackers (*p* < 0.0001), cakes, muffins and pastries (*p* < 0.001), noodles (*p* < 0.001), pasta (*p* = 0.038), rice and rice products (*p* < 0.001), chocolate and sweets/candy (*p* < 0.001), canned seafood (*p* = 0.044), cheese (*p* < 0.001), sauces (*p* < 0.001), spreads and dips (*p* = 0.006), processed meat (*p* < 0.001), and selected snack foods (*p* < 0.001). In contrast, fruit and vegetable juices (*p* < 0.001) and dried seafood (*p* = 0.031) contained significantly lower sodium than the benchmarks (Table 1).

Overall, several food subcategories exceeded the WHO global benchmarks by 0.4% to 60.0%. While biscuits/cookies/crackers exceeded the benchmark by only 2.9%, cakes, muffins, and pastries showed a deviation of 46.8%. Bread and breakfast cereals exceeded by 43.7% and 49.5%, respectively, while frozen Asian dumplings showed the highest deviation at 50.9%. Ready meals and canned seafood also exceeded the benchmarks by 21.9% and 26.9%, respectively. While soup showed a deviation of 0.4%, fish spread, frozen seafood, and cheese exhibited deviations ranging from 27.7% to 44.8% higher than benchmarks, with sauces surpassing by 50.0%. Processed meat shows a 34.2% higher sodium level, while cereal and nut-based snack bars deviate by 34.2% and extruded snacks by 60.8%. Popcorn, potato-based snacks, and salt and vinegar flavoured snacks deviate by 4.3%, 26.6%, and 38.4% respectively. Vegetable-based snacks/chips exceed the benchmark by 9.6%, while wholegrain chips demonstrate a higher deviation of 49.5%.

### Sodium content of packaged foods and beverages in Nigeria, Kenya, and South Africa

Table 3 presents a comparison of the sodium content among subcategories of packaged foods and beverages in Nigeria, South Africa, and Kenya. The study revealed a significant variation in the median sodium content between these countries. Specifically, in the case of Nigeria, approximately 27.9% (*n* = 24) of the food subcategories had a higher sodium content compared to South Africa (*p* < 0.05). Similarly, 26.7% (*n* = 23) of the subcategories in Nigeria exhibited higher sodium content than in Kenya (*p* < 0.05).

Table 4 shows substantial variability in sodium content across packaged food categories lacking WHO benchmarks, with particularly high median levels observed in edible oils (800 mg/100 g), snack packs (736 mg/100 g), and snack foods (474 mg/100 g). In contrast, several beverage categories such as soft drinks (4 mg/100 g) and waters (16 mg/100 g) demonstrated relatively low median sodium levels. The 33rd percentile values provide pragmatic reformulation targets, highlighting significant opportunities for sodium reduction, especially in snack foods, edible oils, and selected dietary-use products where sodium levels and variability (IQR) are notably high.

**Table 3.** Comparison of sodium content by category of packaged foods and beverages between Nigeria, Kenya and South Africa

	Nigeria			South Africa				Kenya			
Food category	Total number of products	Median sodium (mg/100g)	Range (mg/100g)	Total number of products	Median sodium (mg/100g)	Range (mg/100g)	*P*-value (Nigeria vs South Africa)	Total number of Products	Median sodium (mg/100g)	Range (mg/100g)	P-value (Nigeria vs Kenya)
Biscuits/cookies/crackers	1240	273.0	0–40000	526	378	0–2827	**0.001**	412	259	0–1450	**0.022**
Bread	20	568.5	275–4000	174	476	39–2470	**0.007**	169	209	0–600	**<0.001**
Cakes, muffins and pastries	92	385.5	30–1533	147	341	20–1270	**<0.001**	133	200	0–1480	**0.001**
Breakfast cereals	373	198.0	0–6800	376	171	0–4180	**<0.001**	214	112	0–1072	**<0.001**
Couscous	8	9.0	0–38	18	10	0–1262	0.884	1	–	–	–
Miscellaneous cereal and grain products	183	35.0	0–38500	41	5	0–193	**0.024**	154	18	2–900	0.986
Noodles	59	760.0	0–1900	78	470	0–1876	**<0.001**	38	760	9–11940	0.142
Pasta	143	4.0	0–1029	153	4	0–4180	0.127	52	13	0–1480	0.081
Rice and rice products	74	2.0	0–2000	64	8	0–1440	**0.025**	146	0	0–1233	0.885
Chewing gum	46	8.0	0–506	44	1	0–1380	**0.001**	21	0	0–67	0.053
Chocolate and sweets/candy	592	96.0	0–31000	541	74	0–1380	0.907	153	68	0–452	**0.023**
Cough lozenges	1	21.5	22–22	11	0	0–49	0.966	–	–	–	–
Jelly (Jello)	28	12.0	0–120	49	26	0–93	0.232	4	0	0–0	–
Pre–prepared salads and sandwiches	4	360.0	320–1200	82	303	7–818	**<0.001**	–	–	–	–
Ready meals	12	320.0	40–512	156	382	12–2280	0.351	26	503	309–880	**0.040**
Soup	27	236.0	21–5600	270	2017	1–9180	**0.038**	27	240	40–7240	0.533
Canned seafood	117	383.0	80–6360	114	431	0–4430	0.258	–	–	–	–
Frozen seafood	4	488.5	360–1160	94	284	38–670	0.250	–	–	–	–
Seafood and seafood products not otherwise specified	4	1107.0	180–1160	21	449	223–773	0.095	–	–	–	–
Cheese	49	776.0	14–1800	240	554	0–1820	**0.016**	142	720	240–1520	0.620
Cream	19	32.0	0–248	30	36	0–142	0.316	18	68	20–68	**0.012**
Desserts	59	71.1	4–762	69	99	0–601	0.176	15	9	0–6000	**0.037**
Ice cream/edible ices	21	100.0	12–241	55	50	0–179	**0.034**	242	65	4–1600	0.352
Milk	224	56.0	0–1221	253	48	0–822	**<0.000**	177	39	12–396	**<0.001**
Yoghurt/yoghurt drinks	38	36.0	8–113	339	43	0–514	0.176	374	–	–	–
Baby and infant foods	146	146.0	0–367	320	15	0–2050	**<0.001**	20	739	74–215	
Diet drink mixes (meal replacements)	13	89.0	0.4–360	117	347	0–2050	**0.001**	14	–	–	–
Fruit and fruit products	91	5.9	0–5533	466	8	34	**<0.001**	87	7	0–7397	0.176
Herbs and spices	136	200.0	0–35833	202	0	2444	**<0.001**	329	9120	160–23240	0.383
Jam (jelly in the US) and marmalades	64	8.0	0–195	86	10	18	0.928	141	0	0–8000	0.095
Nuts and seeds	154	166.0	0–11000	166	22	140	**<0.001**	172	240	0–3440	**0.002**
Vegetables	365	268.0	0–24000	895	108	353	**0.018**	251	172	0–12000	**<0.001**
Beverage mixes	154	133.0	0–10500	18	205	319	**0.000**	–	–	–	–
Coffee and tea	284	0.0	0–37000	428	0	20	**<0.001**	358	20	0–450	0.512
Cordials (syrups)	7	0.0	0–24	113	56	88	0.600	28	0	0–0	**0.010**
Electrolyte (sports) drinks	7	0.0	0–53	38	42	152	**<0.001**	5	0	0–40	0.500
Energy drinks	48	40.0	0–125	53	35	49	0.585	10	68	0–84	0.360
Fruit and vegetable juices	218	4.0	0–9100	426	6	10	**<0.000**	328	8	0–92	**<0.001**
Soft drinks	111	4.0	0–21000	257	7	11	**0.004**	125	5	0–40	0.105
Waters	25	16.0	0–600	144	2	9	0.183	60	1	0–15	0.076
Mayonnaise/salad dressings	153	535.0	0–1867	183	542		0.579	51	0	220	**0.008**
Sauces	367	680.0	0–22000	704	999	2336	**0.007**	345	1200	3768	**0.002**
Spreads and dips	88	391.0	0–1640	172	386	447	**0.015**	86	438	418	**0.012**
Cooking oils	155	0.0	0–11100	118	0	1	**<0.001**	157	0	0	–
Edible oils	58	470.0	0–1870	88	400	286	0.670	56	195	360	**0.004**
Meat alternatives	2	0.0	0–0	59	748	1219	0.282	16	766	213	0.196
Processed meat	79	912.0	85–7360	486	732	533	0109	167	600	630	**0.001**
Syrups	48	23.0	0–333	–	–	–	–	5	42	42–42	0.029
Potato–based snacks and chips/crisps	137	640.0	40–1887	–	–	–	–	401	480	0–2024	**0.025**
Vitamins and supplements	4	2.0		–	–	–		12	124	108–833	0.615

Data collection years varied by country: Nigeria(2020, 2021, Kenya 2019,) and South Africa (2015, 2016). Variation in collection periods may influence observed differences in sodium content.

**Table 4.** Sodium content of packaged food products and beverages in Nigeria with 33rd percentile targets for categories lacking WHO benchmarks

Main food category	Subcategory	Total number of products	Sodium content		
			Median (mg/100 g)	IQR (mg/100 g)	33^rd^ Percentile
Cereal and grain products	Miscellaneous cereal and grain products	183	35.0	400.0	35
Confectionery	Cough lozenges	1	22	0.0	22
Dairy	Ice cream/edible ices	21	100.0	108.0	56
	Milk	224	56.0	188.0	46
	Probiotic drinks	2	8.0	0.0	8
	Yoghurt/yoghurt drinks	38	36.0	16.0	36
Foods for specific dietary use	Baby and infant foods	146	146.0	167.0	86
	Diet drink mixes (meal replacements)	13	89.0	162.0	54
	Sports products	4	124.0	212.0	88
Non-alcoholic beverages	Beverage mixes	154	133.0	279.0	42
	Breakfast beverages	24	145.0	160.0	125
	Energy drinks	48	40.0	51.5	21
	Fermented beverages (e.g. Kombucha)	2	66.0	0.0	66
	Non-alcoholic beverages not otherwise specified	9	10.0	383.0	10
	Soft drinks	111	4.0	8.2	3
	Waters	25	16.0	18.0	1.3
Edible oils and oil emulsions	Edible oils	58	800	981.0	487
Sugars, honey and related products	Dessert toppings (e.g. caramel sauce)	5	163.0	175.0	154
	Sugar	16	5.2	15.0	5
	Sugars, honey and related products not otherwise specified	6	30.5	80.0	24
	Syrups	48	23.0	166.1	8
Snack foods	Snack foods not otherwise specified	76	474.0	642.0	191
	Snack packs	21	736.0	285.0	657

**Figure 3.**
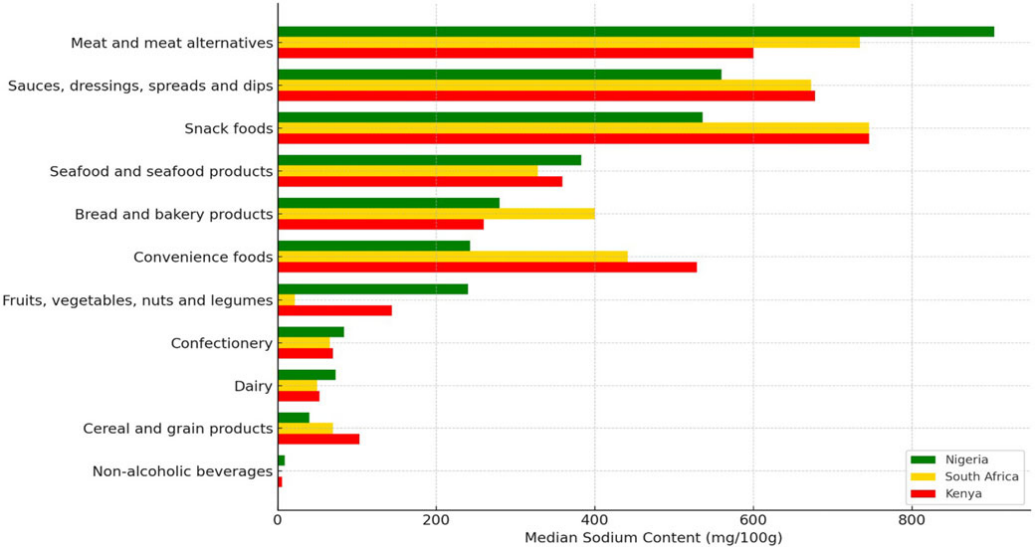
Comparison of median sodium content (mg/100 g) of packaged foods across major food categories in Nigeria, South Africa, and Kenya.

Figure 3 illustrated that across most categories, Nigeria recorded considerably higher sodium levels. In seven out of 11 main food categories (64.0%), Nigeria demonstrated higher sodium content than Kenya (*p* < 0.001), whereas Kenya only reported higher sodium content in four categories (36.0%, *p* < 0.001). For instance, processed meats in Nigeria contain 904 mg/100g of sodium, which is 50.0% more than the median level (600 mg/100g, *p* < 0.001) observed in Kenya. Similarly, condiments and sauces in Nigeria had a median of 559.5 mg/100g of sodium, 17.0% lower than Kenya’s 673 mg/100g (*p* < 0.001). The only exception is the category of Fruits, Vegetables, Nuts, and Legumes, where Kenya has a slightly lower sodium content (144 mg/100g) compared to Nigeria (240 mg/100g) (*p* > 0.05).

Nigeria’s packaged food sodium content also surpassed South Africa’s in some food categories. Nigeria has a higher sodium content than South Africa in six out of 11 main food categories (55.0%, *p* < 0.05). Meat and meat alternatives in Nigeria are particularly high in sodium, containing 904 mg/100g, which is about 19% more than the 734 mg/100g found in South Africa (*p* < 0.05). Seafood products in Nigeria showed a similar trend, with 383 mg/100 g of sodium compared to South Africa’s 328 mg/100 g, a difference that was statistically significant (*p* < 0.05). However, South Africa reported higher sodium content in Bread and Bakery Products (400 mg/100g vs 280 mg/100g in Nigeria, *p* < 0.01) and Cereal and Grain Products (70 mg/100g vs 40 mg/100g in Nigeria).

In Nigeria, the percentage of packaged foods and beverages needing sodium reformulation varied significantly by category, ranging from 11.1% to 100% (Table 5).

**Table 5.** Percentage of subcategory of packaged foods and beverages needing sodium reformation in Nigeria

Main food category	Subcategory	Total number of products	Sodium content		
			Median (mg/100 g)	Percent of food category needing reformulation	WHO global benchmark (mg/100g)
Bread and bakery products	Biscuits/cookies/crackers	1240	273	25.2	265
	Bread	20	569	50.0	320
	Cakes, muffins and pastries	92	386	35.9	205
Cereal and grain products	Breakfast cereals	373	198	23.6	100
Convenience foods	Frozen Asian dumplings/similar products	3	509	100.0	250
	Ready meals	12	320	50.0	250
	Soup	27	236	11.1	235
Seafood and seafood products	Canned seafood	117	383	17.1	280
	Fish spread	2	400	100.0	270
	Frozen seafood	4	489	100.0	270
	Seafood and seafood products not otherwise specified	4	1107	50.0	800
Dairy	Cheese	49	776	59.2	520
Sauces, dressings, spreads and dips	Mayonnaise/salad dressings	153	535	18.3	500
	Sauces	367	680	18.8	340
	Spreads and dips	88	391	15.9	360
	Processed meat	79	912	13.9	600
Snack foods	Extruded snacks	1	1200	100.0	470
	Popcorn	37	491	24.3	470
	Potato-based snacks and hips/crisps	137	640	25.6	470
	Pretzels	2	850	100	760
	Salt and vinegar flavoured snacks (excl. Potato chips)	7	763	42.9	470
	Vegetable-based snacks/chips	9	520	55.6	470
	Wholegrain chips	2	930	100.0	470

## Discussion

This study aimed to assess the sodium content of packaged foods and beverages sold in Nigeria and compared these levels with the WHO Global Sodium Benchmarks, as well as with data from South Africa and Kenya. Findings from this study revealed that the sodium content of packaged foods and beverages in the Nigerian retail market varied across food categories, with more than half of the food categories exceeding WHO Global Sodium Benchmarks. Approximately 40.0% of food categories available in Nigeria were captured in the WHO global sodium benchmark for specific food subcategories, with about 64.0% of subcategories exhibiting higher sodium content than the recommended benchmarks. Additionally, three-quarters of the food products showed higher sodium content in Nigeria compared to WHO benchmarks. This highlight the urgent need for targeted sodium reduction initiatives to align Nigeria’s packaged foods with international health standards and improve public health outcomes. Analysis of sodium levels in Nigerian food subcategories also frequently exceeded those found in both South Africa and Kenya, indicating a widespread issue of elevated sodium content across various Nigerian food categories.

South Africa is the only country in Africa that has successfully implemented national sodium reduction targets and guidelines as part of its efforts to address non-communicable diseases, including hypertension and CVDs. Between 2015 and 2016, South Africa established national sodium benchmarks for packaged foods and beverages as part of its efforts to address the health impacts of excessive sodium consumption. Significant strides have been made in sodium reduction through mandatory measures, with the government implementing maximum limits for sodium content across 13 food categories in 2016, followed by phased enforcement starting in 2016 and further reductions in targets enforced from 2019. This initiative has resulted in a notable decrease of 1.15g/day in salt intake between 2015 and 2019.^(29)^ For Nigeria, the adoption of similar national sodium benchmarks for packaged foods and beverages holds paramount importance in safeguarding public health. These benchmarks would provide vital guidelines and regulations, guiding acceptable levels of sodium content and facilitating collaborative efforts among manufacturers, policymakers, and consumers to reduce excessive sodium dietary intake and mitigate associated health risks.

These results underscore the need for more effective regulations to promote healthier food choices and mitigate potential health risks associated with excessive sodium consumption, similar to other countries with successful sodium reduction initiatives. Several countries have taken significant steps to address salt reduction.^(37)^ Among others, the United Kingdom has implemented a ‘Salt Reduction Programme’ since 2006 which encompassed key elements such as the establishment of voluntary targets to reduce salt content in diverse food categories, advocacy for product reformulation, and the dissemination of information to educate consumers on the potential health consequences linked to excessive salt intake.^(38)^ The United States implemented voluntary sodium reduction targets set by the Food and Drug Administration (FDA); this strategy encompasses collaborative endeavours with public health organisations, industry stakeholders, and scientific experts to enhance awareness, advance education, and endorse initiatives aimed at diminishing dietary sodium intake and enhancing overall public health.^(39)^ However, voluntary targets have not generally been considered effective in the US, as they often lack the enforcement necessary to achieve substantial and consistent reductions in sodium consumption.^(40)^

In contrast, there is a growing readiness in Nigeria and other African countries to implement benchmark-based approaches, which may offer more promise than voluntary schemes such as those used in the UK and USA. Unlike voluntary approaches that rely heavily on industry self-regulation and have shown mixed results, benchmark-based approaches use clear reference values, such as the WHO Global Sodium Benchmarks, that can be translated into enforceable standards. This allows governments to establish mandatory limits for sodium content across food categories, ensuring more consistent reformulation and potentially faster reductions in sodium intake.^(25,41)^ Australia has set sodium reduction targets through the ‘Food and Health Dialogue’ initiative; this strategy includes public awareness campaigns to educate consumers about the health risks associated with high sodium consumption and promote healthier food choices.^(42)^ Finland stands out as a pioneer in salt reduction efforts, implementing comprehensive strategies to reduce salt consumption. Finland’s National Nutrition Council took the pioneering step of launching a salt reduction campaign in the late 1970s, marking one of the earliest efforts by a country to systematically reduce dietary sodium intake among its population. This initiative demonstrates Finland’s proactive stance in tackling the health consequences associated with high sodium consumption and promoting healthier dietary habits.^(9,43)^ Additionally, Colombia has made strides in salt reduction, exemplified by Resolution 2013 which set mandatory maximum sodium targets for 59 categories including snacks, processed meat, dairy, etc.^(44)^ In Chile, a significant sodium reduction initiative is the Law of Food Labelling and Advertising (Law No. 20,606) enacted in 2012. This law regulates sodium content in packaged foods, setting maximum levels for various food categories to combat obesity and promote healthier eating habits. Chile also conducts public health campaigns and educational programmes to raise awareness about the risks of excessive sodium consumption, demonstrating its commitment to improving public health by encouraging healthier dietary habits.^(45)^ These initiatives reflect a global recognition of the importance of salt reduction in promoting public health and preventing related diseases,^(41)^ providing valuable lessons for Nigeria to consider in its own efforts to address sodium consumption. Relating these experiences to our findings, the consistently high sodium levels observed in Nigerian packaged foods highlight the need for a regulatory model closer to South Africa’s mandatory limits, which achieved measurable reductions in salt intake, rather than voluntary schemes such as those in the UK and USA that yielded mixed results. The Nigerian data therefore suggest that adopting enforceable benchmarks, contextualised to the local food supply, could achieve more substantial sodium reduction and provide evidence for future updates of the WHO Global Sodium Benchmarks that currently lack West African representation.

The establishment of national sodium benchmarks for packaged foods and beverages is of paramount importance in safeguarding public health in Nigeria. These benchmarks would serve as essential guidelines and regulations that dictate the acceptable levels of sodium content in such products. By providing reference points for manufacturers, policymakers, and consumers, these benchmarks would enable concerted efforts to reduce excessive dietary sodium intake and mitigate the health risks associated with it. More importantly, the thirty-third percentile is recommended to be used for sodium content targets in various food subcategories in Nigeria where there is no existing WHO sodium benchmarks; This approach aligns with the recommendations provided in the WHO South-East Asia Region Sodium Benchmarks for Packaged Foods. This percentile provides a more achievable target for sodium reduction efforts compared to higher percentiles and can serve as a mandatory target for food manufacturers. The values in the 33rd percentile column indicate the sodium content that 33% of products in each subcategory do not exceed, offering a practical benchmark for sodium reduction in the Nigerian food supply. This approach is based on international best practises,^(36)^ which emphasise setting realistic and attainable targets to encourage compliance and gradual improvement. By adopting the 33rd percentile as a benchmark, Nigeria can effectively promote public health while considering the current state of the food industry. Additionally, using this percentile aligns with strategies employed by other countries to gradually lower sodium content in food products.

Currently, the WHO Global Sodium Benchmarks do not incorporate sodium data from West Africa, leaving a critical evidence gap for the subregion. By providing empirical data from Nigeria, and comparing with Kenya and South Africa, this study helps to fill part of that gap and highlights the importance of regional representation in global benchmark-setting. Incorporating West African data into future updates of the WHO Global Sodium Benchmarks would ensure that targets are more context-specific, feasible for local food environments, and reflective of the unique dietary patterns in the region. This would also support other West African countries that are beginning to consider sodium reduction strategies but lack robust local data to guide their policies.

While this study recommends the adoption of WHO Global Sodium Benchmarks for Nigeria, it is important to note that the WHO report advises that benchmarks should be contextualised to country-specific circumstances. In Nigeria, this could be pursued by setting phased and realistic targets for priority food categories that contribute most to sodium intake, using local market data to adapt thresholds where necessary, and aligning targets with existing regulatory frameworks such as NAFDAC food labelling policies. Engagement with the food industry will also be essential to encourage gradual product reformulation, while consumer education campaigns can help build demand for lower-sodium options. This dual approach using WHO benchmarks as a reference while tailoring implementation to the Nigerian food supply would ensure both feasibility and effectiveness in reducing sodium intake in alignment with the established product-category sodium benchmarks.

The study’s strengths include its comprehensive analysis of sodium content in a broad range of packaged foods and beverages across major Nigerian cities, providing valuable insights into dietary sodium levels. The use of WHO benchmarks as a reference point for comparison is a robust approach, ensuring that the findings are aligned with global standards.

However, the study also has limitations. We reported the percentage of food categories not covered by the benchmarks, but this gap may underestimate sodium exposure from excluded categories. While the study offers comparisons with Kenya and South Africa, it does not account for regional variations within these countries. The secondary data for Kenya and South Africa may also introduce inconsistencies due to differences in data collection methods and periods. Another limitation of this study is the discrepancy in data collection periods across the three countries. While Nigerian data were collected in 2020–2021, the South African dataset dates back to 2015–2016 and the Kenyan dataset to 2019. These differences may affect comparability, as product formulations, labelling practises, and regulatory changes could have occurred during this time. Additionally, sodium estimates were derived from declared nutrition information on food labels rather than direct laboratory analyses, which may introduce reporting errors. Some products lacked complete sodium information, potentially leading to bias. Although data cleaning and quality assurance procedures were undertaken to minimise errors, reliance on secondary label data may still affect accuracy.

## Conclusion

The findings contribute to our understanding of the sodium landscape in Nigeria and can inform policymaking and interventions aimed at improving public health through better food choices and sodium reduction strategies. Overall, these findings highlight the need for continued monitoring and regulation of sodium levels in specific subcategories of foods to promote healthier choices and align with WHO Global Sodium Benchmarks. With nearly two-thirds of Nigerian food subcategories exceeding WHO benchmarks and sodium levels surpassing those in South Africa and Kenya across most major categories, the study findings highlight the urgency of implementing targeted sodium reduction measures. By embracing the WHO’s more rigorous and comprehensive sodium benchmarks, the Nigerian government, in collaboration with key agencies such as the National Agency for Food and Drug Administration and Control, has the potential to significantly enhance the health outcomes and benefits of the sodium reformulation programme. The finding emphasises the necessity for prompt discussions between public health authorities, government representatives, and food companies, in collaboration with experts from scientific societies. There is a need for a comprehensive strategy for reformulating food products that are not aligned with the specific targets outlined by the WHO guidelines. We recommend the adoption of the WHO Global Sodium Benchmarks where available, and if no benchmark exists, set the target at the 33rd percentile of current products in Nigeria. This approach would provide clear and actionable guidelines to drive sodium reduction efforts. Implementing stringent sodium benchmarks could not only help in aligning Nigerian food products with international standards but also yield long-term benefits by reducing healthcare costs associated with diseases related to high dietary sodium intake, potentially resulting in economic savings and a healthier workforce.

## Data Availability

The FoodSwitch database used in this study is not publicly available due to institutional restrictions. Access may be granted upon reasonable request to The George Institute for Global Health, subject to approval and data-sharing agreements.
